# Overcoming Access Barriers for Veterans: Cohort Study of the Distribution and Use of Veterans Affairs’ Video-Enabled Tablets Before and During the COVID-19 Pandemic

**DOI:** 10.2196/42563

**Published:** 2023-01-26

**Authors:** Zainub Dhanani, Jacqueline M Ferguson, James Van Campen, Cindie Slightam, Josephine C Jacobs, Leonie Heyworth, Donna Zulman

**Affiliations:** 1 Department of Health Policy Stanford School of Medicine Stanford, CA United States; 2 Center for Innovation to Implementation Veterans Affairs Palo Alto Health Care System Menlo Park, CA United States; 3 Division of Primary Care and Population Health Stanford School of Medicine Palo Alto, CA United States; 4 Health Economics Resource Center Veterans Affairs Palo Alto Health Care System Menlo Park, CA United States; 5 Department of Veterans Affairs Central Office Office of Connected Care/Telehealth Services Veterans Health Administration Washington, DC United States; 6 Department of Medicine University of California San Diego Health System San Diego, CA United States

**Keywords:** COVID-19, veterans, health care access, video-based care, telehealth, barriers to care, telemedicine, veteran's association, health disparity, sociodemographic, virtual health

## Abstract

**Background:**

During the COVID-19 pandemic, as health care services shifted to video- and phone-based modalities for patient and provider safety, the Veterans Affairs (VA) Office of Connected Care widely expanded its video-enabled tablet program to bridge digital divides for veterans with limited video care access.

**Objective:**

This study aimed to characterize veterans who received and used US Department of VA–issued video-enabled tablets before versus during the COVID-19 pandemic.

**Methods:**

We compared sociodemographic and clinical characteristics of veterans who received VA-issued tablets during 6-month prepandemic and pandemic periods (ie, from March 11, 2019, to September 10, 2019, and from March 11, 2020, to September 10, 2020). Then, we examined characteristics associated with video visit use for primary and mental health care within 6 months after tablet shipment, stratifying models by timing of tablet receipt.

**Results:**

There was a nearly 6-fold increase in the number of veterans who received tablets in the pandemic versus prepandemic study periods (n=36,107 vs n=6784, respectively). Compared to the prepandemic period, tablet recipients during the pandemic were more likely to be older (mean age 64 vs 59 years), urban-dwelling (24,504/36,107, 67.9% vs 3766/6784, 55.5%), and have a history of housing instability (8633/36,107, 23.9% vs 1022/6784, 15.1%). Pandemic recipients were more likely to use video care (21,090/36,107, 58.4% vs 2995/6784, 44.2%) and did so more frequently (5.6 vs 2.3 average encounters) within 6 months of tablet receipt. In adjusted models, pandemic and prepandemic video care users were significantly more likely to be younger, stably housed, and have a mental health condition than nonusers.

**Conclusions:**

Although the COVID-19 pandemic led to increased distribution of VA-issued tablets to veterans with complex clinical and social needs, tablet recipients who were older or unstably housed remained less likely to have a video visit. The VA’s tablet distribution program expanded access to video-enabled devices, but interventions are needed to bridge disparities in video visit use among device recipients.

## Introduction

### Background

Many US veterans experience geographic and health-related barriers to accessing care. Nearly 1 in 3 of the approximately 9 million veterans enrolled in the US Department of Veterans Affairs (VA) live in rural, highly rural, or insular areas, and many more experience transportation challenges and financial insecurity that impede their use of VA services [1-3]. The COVID-19 pandemic magnified these barriers and introduced new challenges, such as social isolation. In addition, shelter-in-place orders early in the pandemic led to decreased public transportation options, increased costs for private transportation, and financial strain due to job loss.

VA was in a strong position to address these pervasive access barriers as an early adopter of care provided via phone or video [4], particularly within mental health and primary care clinics. In addition, VA’s Office of Connected Care and Office of Rural Health had previously launched an initiative to distribute video-enabled tablets to veterans with access barriers who did not have a suitable device or data plan. This program, launched in 2016, was designed to expand video teleconferencing into veterans’ homes through tablet-enabled secure video technology [3]. Devices are available for veterans without access to the necessary technology for video teleconferencing and for those who also face geographic, clinical, and social barriers to in-person care.

For many veterans facing barriers to video care during the pandemic, VA’s video-enabled tablet distribution program offered a potential solution. As COVID-19 increased demand for video-based alternatives to in-person care, VA rapidly expanded its tablet distribution program [4]. The goal of this intensive tablet distribution effort was to facilitate video care and thereby reduce risks of COVID-19 exposure for patients and providers, preserve facility resources for higher-acuity patients, and support continuity of care for patients who might otherwise be unable to access care [4-7]. However, it was unclear how this rapid transition to video care would influence individuals with complex medical and social needs [8], including individuals who often experience “digital divide” disparities in accessing video care, including older adults, certain racial minority groups, and individuals who have low-income or reside in rural locations [8-10].

### Objectives

To inform VA’s efforts to bridge the digital divide, we sought to characterize veterans who received video-enabled tablets from VA before and during the COVID-19 pandemic. At the start of the pandemic, frontline VA health care providers were instructed to identify and reach out to high-risk individuals; however, determining which veterans should be contacted was left up to the providers’ discretion [4]. We examined changes in the sociodemographic and clinical characteristics of tablet recipients before and after the pandemic began and analyzed the association between these characteristics and subsequent use of video visits for clinical care. Ultimately, our project aimed to contribute to understandings of the impact of the pandemic on telehealth access disparities among veterans enrolled in the VA tablet program.

## Methods

### Data Extraction and Study Population

We extracted encounter and patient level data from VA’s Corporate Data Warehouse, a repository for VA electronic health records [11]. We defined 2 cohorts of veterans who received tablets from VA during 6-month windows spanning similar seasons: (1) a prepandemic cohort comprising those who received tablets between March 11, 2019, and September 10, 2019; and (2) a pandemic cohort of individuals who received tablets between March 11, 2020, and September 10, 2020 (after COVID-19 was officially designated as a pandemic by the World Health Organization) [12]. We then assessed the use of VA health care services for each veteran for 6 months after tablet shipment.

We excluded patients who identified as nonveterans, living on American territory islands, missing a zip code (needed for urban or rural classification) or VA priority enrollment group (described below), and those whose date of death preceded tablet shipment. The analytic cohort comprised 42,891 veterans (6784 prepandemic recipients and 36,107 pandemic recipients).

### Classification of VA Outpatient Health Care Encounters

All outpatient encounters were classified by type of care and modality using VA’s Managerial Cost Accounting Stop Codes as described in detail previously [6]. In short, these 3-digit nationally standardized numerical codes characterize outpatient VA clinic encounters and identify the work group responsible for providing the documented clinical services [6]. The type of care included 4 mutually exclusive categories: primary care, specialty care, mental health care, and other care. Other care comprised rehabilitation care, emergency care, and diagnostic or ancillary care. Care modality categories included in-person care, phone care, video care, or supplementary remote care (including remote patient monitoring, telehealth care coordination, and home telehealth) [13].

### Outcome

The primary outcome of interest was any video care encounter after a veteran received a VA-issued tablet. Regression analyses assessed characteristics associated with video care use in primary and mental health care encounters given these are the 2 most common types of VA care delivered via video and comprised 81% and 79% of all video encounters for the prepandemic and pandemic cohorts, respectively [4,6].

To limit encounters to those that occurred when a veteran had their VA-issued tablet, we excluded encounters that occurred within 7 days after tablet shipment. This time frame was selected to account for variation in shipping and setup time and because VA contacts tablet recipients 7 days after shipment to assist with tablet setup [4].

### Patient Sociodemographic and Clinical Characteristics

Patient-level data consisted of sociodemographic and clinical characteristics of veterans who were shipped tablets during the prepandemic and pandemic study periods. Race and ethnicity were documented based on the veterans’ most frequently reported identification in patient health records [14]. Missing data on race, ethnicity, and marital status were treated as distinct categories. Urban and rural definitions were derived from 2010 US Census Bureau criteria based on a veteran’s home zip code [15]. Highly rural areas were areas with a population density fewer than 7 people per square mile. History of housing instability was defined using outpatient stop codes reflecting the use of housing instability services and VA diagnosis codes. The 18 regional systems of care, known as Veterans Integrated Service Network (VISN), served as a proxy for geographic region [16]. Given the small cell number for VISN 5, representative of the VA Capitol Health Care Network, we combined VISN 5 with VISN 6, which represents the VA Mid-Atlantic Health Care Network. Criteria for the 28 chronic conditions and diagnoses used to determine multimorbidity status (Multimedia Appendix 1) as well as criteria for being categorized as having mental health conditions (Multimedia Appendix 2) were based on previous studies of VA video and phone care [6,17-20].

Analyses also included veterans’ VA priority status as indicated by the VA priority-based enrollment system. VA enrolls veterans into 1 of 8 mutually exclusive groups based on their service-connected disability rating, income, recent military service, and other factors. We generated 4 categories of priority status: low income, low or moderate disability, high disability, or no special enrollment (Multimedia Appendix 3) [14].

To account for the association between past and future use of VA health care, we assessed the use of VA health care in the 365 days prior to and excluding the date of tablet shipment for all patients in both cohorts. We categorized health care use as low use, moderate use, or high use corresponding to 0th to 25th, 25th to 75th, and 75th to 100th percentiles. Prior in-person and video visits were dichotomized (eg, prior video use: yes or no). Prior use of remote patient monitoring, specialty care, and rehabilitation care was combined into one category (*other*) due to small numbers.

### Statistical Analyses

We calculated frequencies and percentages of patient sociodemographic and clinical characteristics across the 2 cohorts. We then examined the average number of mental health or primary care encounters via video for both cohorts in the 6 months after individuals received their tablets. To assess the association between sociodemographic characteristics of tablet recipients and video care use, we used generalized linear models to predict the likelihood of having a video mental health or primary care visit within 6 months of tablet receipt for each cohort (before the pandemic and during the pandemic). We adjusted for patient demographics, clinical characteristics, region, and previous use of VA health care. We report adjusted incident risk ratios (RRs) and 95% CIs for all models.

Additional analyses examined the relative proportion of encounters by modality (ie, in-person, phone, video, or remote monitoring) and care category (ie, primary, mental health, specialty, or other) within each cohort. We also assessed the time between tablet shipment and recipients’ first mental health or primary care video visit as well as the total number of mental health or primary care video encounters recipients completed within 6 months of tablet shipment for both prepandemic and pandemic cohorts. All statistical analyses and graphical output were completed using Stata statistical software (version 17; StataCorp, LLC).

### Ethical Considerations

This evaluation was completed as part of a VA Quality Enhancement Research Initiative and was designated as nonresearch quality improvement by VA’s Office of Rural Health, local institutional review board, and VA Research Administration.

## Results

There was a nearly 6-fold increase in VA tablet recipients between the prepandemic study period in 2019 (n=6784) and the pandemic study period in 2020 (n=36,107; Table 1). There were notable changes in the demographics of tablet recipients over this time. Overall, the pandemic cohort (compared to the prepandemic cohort) was older (mean age 64.4 vs 58.8 years), and a higher proportion was male (32,070/36,107, 88% vs 5620/6784, 82.8%). There were modest changes in race and ethnicity between the 2 cohorts (eg, the proportion of Black or African American tablet recipients increased from 1481/6784, 21.8% to 9220/36,107, 25.5% and Hispanic/Latinx recipients decreased from 409/6784, 6% to 1638/36,107, 4.5%). Compared to the prepandemic period, there was also an increase in the proportion of pandemic tablet recipients who were urban-dwelling (24,504/36,107, 67.9% vs 3766/6784, 55.5%) and had a history of housing instability (8633/36,107, 23.9% vs 1022/6784, 15.1%). Pandemic versus prepandemic recipients were less likely to be documented as married in the electronic health records (14,713/36,107, 40.7% vs 3262/6784, 48.1%, respectively). Tablet recipients with a VA priority enrollment group related to low income were more heavily represented during the pandemic versus before the pandemic (9309/36,107, 25.8% vs 1272/6784, 18.8%, respectively) but less represented by a VA priority status related to high disability (18,142/36,107, 50.2% vs 4013/6784, 59.2%, respectively). Pandemic tablet recipients also had a slightly higher mean number of chronic conditions versus prepandemic recipients (7.6 vs 7.3 chronic conditions, respectively), and fewer had a mental health condition (26,948/36,107, 74.6% vs 5421/6784, 79.9%, respectively).

Tablet recipients in the pandemic period had over twice as many video visits in the 6 months after tablet receipt as prepandemic tablet recipients (4.4 and 1.7 mental health or primary care encounters on average, respectively). Conversely, pandemic tablet recipients had fewer average in-person care encounters compared to prepandemic tablet recipients (4.9 and 7.5 mental health or primary care encounters, respectively). The overall trends in prior use for all tablet recipients were very similar for in-person and primary care encounters. Other baseline use patterns are presented in Table 2.

**Figure 1:**
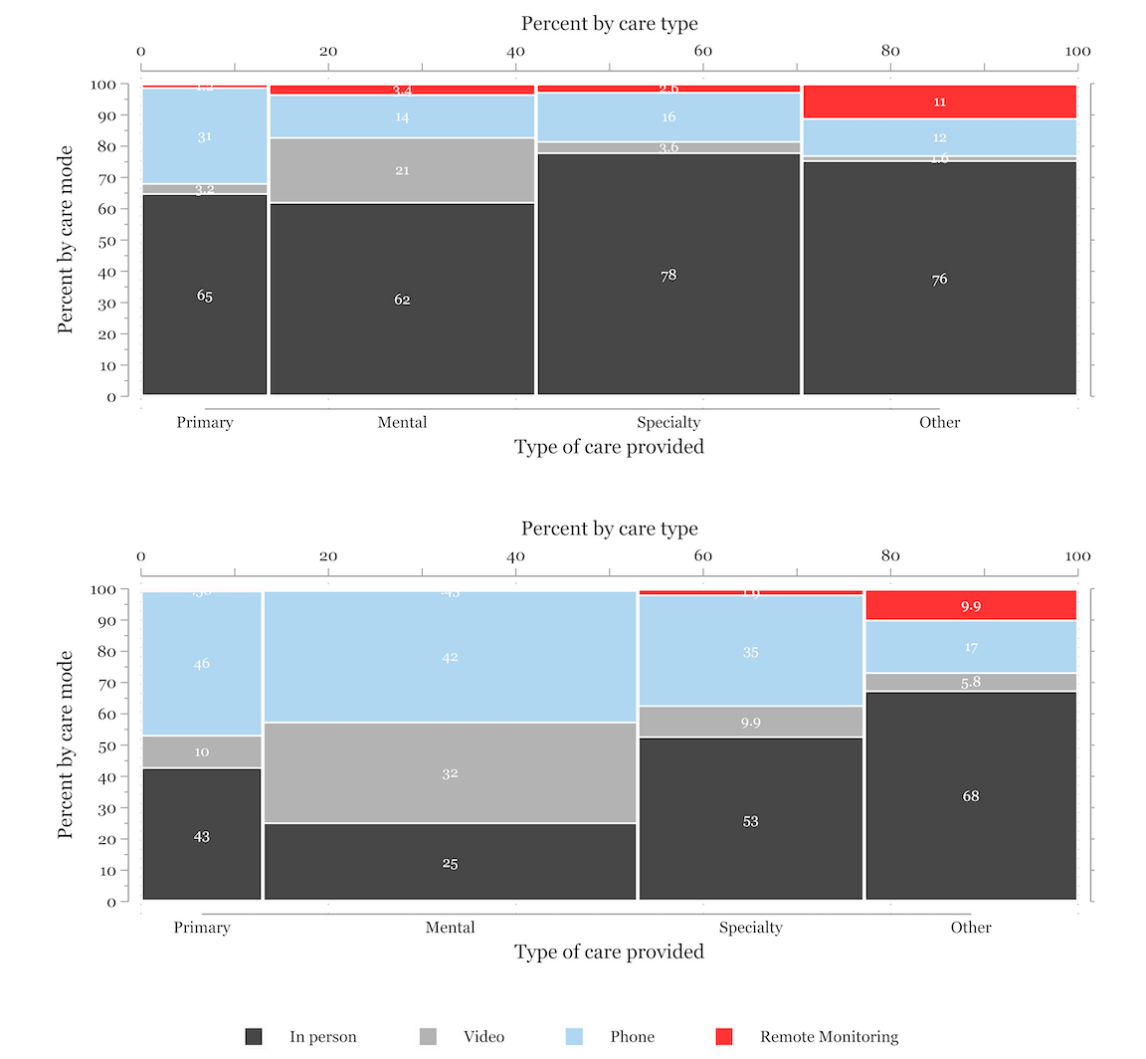
Encounter patterns in 6 months after tablet receipt, by specialty and modality, for veterans in the (top panel) prepandemic (n=191,143 encounters) and (bottom panel) pandemic (n=1,121,898 encounters) cohorts.

Figure 1 illustrates patterns of encounters after tablet receipt, by specialty and care modality, among the prepandemic cohort (191,143 encounters) and pandemic cohort (1,121,898 encounters). There were notable spikes in phone care during the pandemic, larger than the increases in video care use compared to before the pandemic. Overall, video encounters comprised 16.5% (216,232/1,313,041) of total combined encounters for both cohorts within 6 months of tablet shipment. The pandemic cohort had a lower proportion of encounters in person (492,534/1,121,898, 43.9% vs 135,723/191,143, 71%) and more encounters by phone (394,942/1,121,898, 35.2% vs 30,609/191,143, 16%) and video (201,231/1,121,898, 17.9% vs 15,001/191,143, 7.9%) compared to the prepandemic cohort. Both before and during the pandemic, mental health care had the largest percentage of encounters among the 4 categories of care types, although mental health care encounters accounted for an additional 12% of total encounters for the pandemic cohort compared to the prepandemic cohort. Additional details about tablet use patterns (eg, distribution of time from tablet shipment until first video encounter and distribution of total video encounters per recipient) can be found in Multimedia Appendices 4 and 5.

Adjusted models stratified by prepandemic and pandemic tablet receipt (Table 3) showed that users of video care in both groups were significantly more likely to be younger, stably housed, and have a mental health condition (ie, the CIs exclude the null value 1). Compared to those aged 18-44 years, those 65 years or older in the pandemic and prepandemic cohorts were 17% (RR 0.83, 95% CI 0.79-0.87) and 18% (RR 0.82, 95% CI 0.73-0.92) less likely, respectively, to use video primary or mental health care. Veterans with a history of housing instability had 8% (RR 0.92, 95% CI 0.88-0.95) and 19% (RR 0.81, 95% CI 0.72-0.91) lower likelihoods of video care use in the pandemic and prepandemic cohorts, respectively. Women in both cohorts had a 4% (prepandemic: RR 1.04, 95% CI 0.95-1.14; pandemic: RR 1.04, 95% CI 1.00-1.08) increased likelihood of video care use, but the CIs included the null in the prepandemic model. Both rural and highly rural veterans were 13% (rural: RR 1.13, 95% CI 1.04-1.22; highly rural: RR 1.13, 95% CI 0.95-1.33) more likely than urban veterans to use video care in the prepandemic cohort, although the CIs were wide for the highly rural group. There were no differences in likelihood between rurality groups in the pandemic cohort. There were minor, statistically nonsignificant, and likely clinically insignificant differences in likelihood of having a video visit based on race, ethnicity, multimorbidity, and VA priority group.

**Table 1:** Characteristics of study population and demographics of tablet recipients before and during the COVID-19 pandemic (N=42,981).

Characteristic		Prepandemic tablet recipients (from March 11, 2019, to September 10, 2019; n=6784, 15.8%), n (%)	Pandemic tablet recipients (from March 11, 2020, to September 10, 2020; n=36,107, 84.2%), n (%)
**Age group (years)**			
	18-44	1407 (20.7)	3781 (10.5)
	45-64	2646 (39)	12,828 (35.5)
	65+	2731 (40.3)	19,498 (54)
**Race**			
	American Indian or Alaska Native	113 (1.7)	433 (1.2)
	Asian	36 (0.5)	235 (0.7)
	Black or African American	1481 (21.8)	9220 (25.5)
	Native Hawaiian or Pacific Islander	66 (1)	365 (1)
	Unknown	351 (5.2)	1653 (4.6)
	White	4737 (69.8)	24,201 (67)
**Ethnicity**			
	Hispanic/Latino	409 (6)	1638 (4.5)
	Not Hispanic/Latino	6158 (90.8)	33,394 (92.5)
	Unknown	217 (3.2)	1075 (3)
**Gender**			
	Male	5620 (82.8)	32,070 (88.8)
	Female	1164 (17.2)	4037 (11.2)
**Marital status**			
	Single (or divorced or widowed)	3457 (51)	21,073 (58.4)
	Married	3262 (48.1)	14,713 (40.7)
	Unknown	65 (1)	321 (0.9)
**Rurality**			
	Urban	3766 (55.5)	24,504 (67.9)
	Rural	2656 (39.2)	10,458 (29)
	Highly rural	362 (5.3)	1145 (3.2)
**History of housing instability**			
	No	5762 (84.9)	27,474 (76.1)
	Yes	1022 (15.1)	8633 (23.9)
**Number of chronic conditions**			
	0-3	962 (14.2)	4823 (13.4)
	4-6	2179 (32.1)	10,839 (30)
	7-9	1949 (28.7)	10,428 (28.9)
	10+	1694 (25)	10,017 (27.7)
**Any mental health condition(s)**			
	No	1363 (20.1)	9159 (25.4)
	Yes	5421 (79.9)	26,948 (74.6)
**VA^a^ priority enrollment group**			
	No special enrollment	447 (6.6)	2583 (7.2)
	Low income	1272 (18.8)	9309 (25.8)
	Low or moderate disability	1052 (15.5)	6073 (16.8)
	High disability	4013 (59.2)	18,142 (50.2)

^a^VA: Veterans Affairs.

**Table 2:** Characteristics of video use and prior Veterans Affairs (VA) health care use for tablet recipients before and during the COVID-19 pandemic.

Characteristic		Prepandemic tablet recipients (from March 11, 2019, to September 10, 2019; n=6784), n (%)		Pandemic tablet recipients (from March 11, 2020, to September 10, 2020; n=36,107), n (%)
**Had a primary care or mental health video encounter in the 6 months after tablet receipt**				
	No	3789 (55.9)	15,017 (41.6)	
	Yes	2995 (44.2)	21,090 (58.4)	
**Number of primary care or mental health video encounters in the 6 months after tablet receipt**				
	0	3789 (55.9)	15,017 (41.6)	
	1-2	1544 (22.8)	9253 (25.6)	
	3-5	743 (11)	4570 (12.7)	
	6-10	428 (6.3)	3083 (8.5)	
	11-25	277 (4.1)	2891 (8)	
	26-50	3 (0.04)	915 (2.5)	
	50+	0 (0)	378 (1.1)	
**Prior in-person encounters^a^**				
	Low (1-13)	1729 (26.1)	9330 (25.8)	
	Moderate (14-54)	3437 (50.7)	17,528 (48.5)	
	High (>54)	1578 (23.3)	9249 (25.6)	
**Prior video encounters^a^**				
	None	5971 (87.2)	26,583 (73.6)	
	Any	867 (12.8)	9524 (26.4)	
**Prior phone encounters^a^**				
	Low (0-2)	1963 (28.9)	4504 (12.5)	
	Moderate (3-10)	2794 (41.2)	14,487 (40.1)	
	High (<10)	2027 (29.9)	17,116 (47.4)	
**Prior primary care encounters^a^**				
	Low (0-3)	1586 (23.4)	8088 (22.4)	
	Moderate (4-10)	3616 (53.3)	19,543 (54.1)	
	High (>10)	1582 (23.3)	8476 (23.5)	
**Prior mental health encounters^a^**				
	None	1502 (22.1)	9449 (26.2)	
	Low (1-6)	2227 (33.6)	8532 (23.6)	
	High (>6)	3005 (44.3)	18,126 (50.2)	

^a^Prior encounters are based on encounters within the 365 days prior to tablet receipt calculated using encounters in all care specialties (not only primary care and mental health care encounters).

**Table 3:** Adjusted incidence risk ratios and 95% CIs for primary and mental health Veterans Affairs (VA) video care use within 6 months of tablet receipt among veterans who received their tablets before or during the COVID-19 pandemic.

Characteristics		Model 1: prepandemic cohort^a^ (n=6784), risk ratio (95% CI)	Model 2: pandemic cohort^b^ (n=36,107), risk ratio (95% CI)
**Age group (years)**			
	18-44	1.00	1.00
	45-64	0.97 (0.88-1.07)	0.91 (0.87-0.96)
	65+	0.82 (0.73-0.92)	0.83 (0.79-0.87)
**Race**			
	American Indian or Alaska Native	1.01 (0.77-1.33)	0.96 (0.85-1.08)
	Asian	1.20 (0.80-1.79)	0.97 (0.82-1.14)
	Black or African American	0.94 (0.85-1.04)	0.99 (0.96-1.00)
	Native Hawaiian or Pacific Islander	1.05 (0.75-1.48)	0.97 (0.85-1.11)
	Unknown	0.99 (0.83-1.19)	0.98 (0.91-1.05)
	White	1.00	1.00
**Ethnicity**			
	Not Hispanic/Latino	1.00	1.00
	Hispanic/Latino	0.95 (0.82-1.11)	1.01 (0.94-1.07)
	Unknown	1.03 (0.82-1.29)	1.00 (0.92-1.10)
**Gender**			
	Male	1.00	1.00
	Female	1.04 (0.95-1.14)	1.04 (1.00-1.08)
**Rurality**			
	Urban	1.00	1.00
	Rural	1.13 (1.04-1.22)	1.00 (0.97-1.03)
	Highly rural	1.13 (0.95-1.33)	1.00 (0.92-1.08)
**History of housing instability**			
	No	1.00	1.00
	Yes	0.81 (0.72-0.91)	0.92 (0.88-0.95)
**Number of chronic conditions**			
	0-3	1.00	1.00
	4-6	1.01 (0.90-1.14)	0.99 (0.94-1.04)
	7-9	0.98 (0.86-1.12)	1.00 (0.95-1.05)
	10+	0.94 (0.80-1.10)	1.00 (0.94-1.06)
**Any mental health condition(s)**			
	No	1.00	1.00
	Yes	1.34 (1.14-1.57)	1.12 (1.06-1.18)
**VA^c^ priority enrollment group**			
	No special enrollment	1.00	1.00
	Low income	1.02 (0.85-1.23)	0.96 (0.90-1.02)
	Low or moderate disability	1.04 (0.86-1.25)	0.98 (0.92-1.05)
	High disability	1.13 (0.96-1.34)	1.03 (0.97-1.09)

^a^Prepandemic cohort comprises all veterans who received tablets between March 11, 2019, and September 10, 2019; these veterans used their tablets for a maximum of 6 months after receiving their tablets, with all included use occurring on or before March 10, 2020.

^b^Pandemic cohort comprises all veterans who received tablets between March 11, 2020, and September10, 2020; these veterans used their tablets for a maximum of 6 months after receiving their tablets, with all included use occurring on or before March 10, 2021.

^c^VA: Veterans Affairs.

## Discussion

### Principal Findings

Our results demonstrated that tablet distribution patterns changed significantly during the COVID-19 pandemic, with higher clinical and social need veterans being more likely to receive a tablet; however, barriers remain in increasing use of video care among these veterans. VA’s rapid expansion of the tablet distribution program during the COVID-19 pandemic resulted in a nearly 6-fold increase in the number of veterans who received a tablet during 6-month comparison periods. This evaluation of the tablet distribution program highlights changes in sociodemographic and clinical characteristics of tablet recipients before and during the pandemic. Overall, findings suggest that VA’s tablet distribution program facilitated critical access to video care compatible devices for veterans with high levels of clinical and social needs, although a digital divide persisted in terms of how the devices were used.

The fact that a higher proportion of tablet recipients during the pandemic were older and had low income (based on VA priority status) suggests that the expansion of the tablet distribution program, alongside the implementation of interventions aimed at facilitating technology literacy, may have facilitated a narrowing of the digital divide for these groups during the pandemic. Older veterans and veterans with a history of housing instability are groups that often face barriers to telemedicine and thus may have been more heavily targeted for tablet distribution due to higher risks of severe COVID-19 illness in these populations [21]. Additionally, higher distribution of tablets to urban veterans during the pandemic may reflect the largely urban COVID-19 infection hotspots in the United States during the first 6 months of the pandemic, driving demand for video-based services in these settings [22]. Tablet recipients during the first 6 months of the pandemic also had higher numbers of chronic conditions on average, potentially due to greater reliance on VA and greater demand for clinical care early in the pandemic.

Based on encounter data, tablet recipients during the pandemic (vs the prepandemic period) were more likely to have a video encounter within 6 months of receiving their tablet, although phone visits remained the most common form of telehealth among pandemic recipients, in line with a growing body of evidence illustrating this pattern across multiple countries [23]. Veterans also had a higher average number of video encounters during that time period and had shorter average windows of time between tablet shipment and a recipient’s first video visit following shipment. Although these analyses were not adjusted for confounders such as patient characteristics, these associations may be due to several factors, including but not limited to VA limitations on in-person visits to reduce COVID-19 exposures (supported by corresponding decreases in the number of in-person visits), as well as broader increases in programming and resource allocation toward video or phone care during the pandemic. Comparisons of the time to first video visit for tablet recipients and veterans with their own devices could provide meaningful insights on the impact of device provision of video care uptake. Since the time of this evaluation, VA has implemented an attestation to encourage providers who order a tablet for a veteran to conduct a video visit within 90 days of referral.

Findings from this study expand on previous analyses that showed that VA patients aged older than 45 years or those with a history of housing instability had a reduced likelihood of video care use during the first 3 months pandemic, despite these populations having higher clinical and social needs [6]. We found that tablet recipients aged older than 45 years had significantly lower likelihoods of video care use during the pandemic. Given that many older veterans may have first attempted video care during the pandemic due to limitations on in-person care, barriers to overcoming the learning curve of digital literacy necessary for video care may have contributed to decreased use among this population. Veterans with a history of housing instability had lower use of video care both before and during the pandemic compared to stably housed veterans receiving tablets. As previous research has indicated unique barriers for subpopulations of pandemic tablet recipients with a history of housing instability [14], the findings in our study further highlight the need for additional tailored resources when addressing barriers faced by this population.

We also identified use patterns that may have been influenced by the social impacts and hotspot trajectory of the pandemic. Despite being proportionally less likely than men to receive a tablet during the pandemic, recipients who were women had an increased likelihood to use video care. Although this is consistent with previous studies showing that women veterans are higher users of all types of video care [6], these trends may be indicative of higher likelihoods of women preferring video or phone care or being more likely to have caregiving priorities during the pandemic [24]. Rural and highly rural prepandemic tablet recipients had increased likelihoods of use of video care compared to urban recipients. However, among the pandemic cohort, the likelihoods of use were equivalent across all rurality groups. This may reflect a shift away from distance being a primary motivator for video care use during the pandemic. Given the higher proportion of urban tablet recipients during the pandemic, these findings may also reflect the lower relative COVID-19 infection risks for rural and highly rural veterans early in the pandemic, whereas prior to the pandemic, rural and highly rural veterans may have been more likely to use video care as a means of circumventing barriers to in-person care [4].

One point of concern is the potential for video care to increase disparities in health care access among racial and ethnic groups who may have health care access barriers prior to the pandemic [8-10,25]. In our evaluation, we found no statistically significant differences in video use among all racial and ethnic groups during the pandemic. However, the relatively small numbers of Asian, American Indian or Alaska Native, and Native Hawaiian or Pacific Islander tablet recipients limit our conclusions. Additionally, the limited available racial and ethnic categories (ie, no multiracial category, no detailed ethnicity, or no regions of national origin) are unlikely to reflect the structural racism that veterans of racial and ethnic minority groups experience.

Ultimately, findings suggest a need for targeted interventions aimed at increasing uptake of video care among older and unstably housed veterans. Future research should also consider the unique impact of the COVID-19 pandemic and the expansion of video care on health care access for specific subgroups of veterans as well as potential areas for program improvement for veterans with higher clinical or social needs. Additionally, our analyses showed that nearly one-third of tablet recipients included in this study did not have any video visits within 6 months of receiving their tablet. Further investigations into demographic trends associated with video care disuse and methods of increasing video care uptake among tablet recipients would provide valuable information for further improvements to the tablet program.

There are several limitations to this study that should be noted. Although findings indicate that VA’s tablet distribution program advanced accessibility of video care among veterans both before and during the pandemic, video visits accounted for only a minority of all encounters; future work should consider the balance of video, phone, and in-person care. In addition, it would be of value to understand the impact of tablets of different specialties of care during the pandemic. Although the impact of tablets on mental health video care use during the pandemic has been described [26], future research to similarly assess the impact of tablet distribution on primary and specialty care video visit use would provide additional insights on how tablets affect veterans’ access to care. Another limitation of this evaluation is the absence of confirmation that video visits took place via VA-issued tablets, although a criterion for tablet receipt is that a veteran does not have a video-enabled device with a suitable data plan. Further, there are challenges in identifying failed video visits that were converted to phone visits, making it difficult to fully assess the degree to which tablets influenced video care use. Future research should examine the factors that influence successful video visit completion, particularly as VA expands support services for veterans who experience difficulties using video care technology.

### Conclusions

In summary, this large, nationally representative evaluation characterized the reach and impact of VA’s effort to scale up distribution of video-enabled tablets to veterans during the COVID-19 pandemic. We found that the pandemic was associated with striking changes in the characteristics of tablet recipients and use. Notably, veterans with indicators of increased clinical and social need were more likely to receive tablets during the pandemic, although this need did not translate into greater use of video care. These findings suggest that health systems aiming to enhance access through video-capable devices should consider organizational, external, and patient-level factors influencing the uptake of video care.

## Appendix

Multimedia Appendix 1 List of chronic conditions.

Multimedia Appendix 2 List of mental health conditions.

Multimedia Appendix 3 Description of Veterans Affairs enrollment priority group.

Multimedia Appendix 4 Time in weeks between tablet shipment and first primary or mental health care video visit for the prepandemic and pandemic cohorts of tablet recipients among those with at least one video visit. The prepandemic cohort includes all veterans who received tablets between March 11, 2019, and September 10, 2019, and the pandemic cohort includes all veterans who received tablets between March 11, 2020, and September 10, 2020.

Multimedia Appendix 5 Distribution of the total number of video primary care and mental health care video visits in the 6 months after tablet shipment for the prepandemic and pandemic cohorts of tablet recipients among those with at least one video visit. The prepandemic cohort includes all veterans who received tablets between March 11, 2019, and September 10, 2019, and pandemic cohort includes all veterans who received tablets between March 11, 2020 and September 10, 2020.

## Abbreviations

RR: risk ratio
VISN: Veterans Integrated Service Network
VA: Veterans Affairs

## Data Availability

Due to US Department of Veterans Affairs (VA) regulations and our ethics agreements, the analytic data sets used for this study are not permitted to leave the VA firewall without a Data Use Agreement. This limitation is consistent with other studies based on VA data. However, VA data are made freely available to researchers with an approved VA study protocol. For more information, please visit https://www.virec.research.va.gov or contact the VA Information Resource Center at VIReC@va.gov.
