# Supplementary material for: Overcoming Access Barriers for Veterans: Cohort Study of the Distribution and Use of Veterans Affairs’ Video-Enabled Tablets Before and During the COVID-19 Pandemic
Source: J Med Internet Res. 2023 Jan 26;25:e42563. doi: 10.2196/42563 (PMC9912147; doi:10.2196/42563)
Supplement: Multimedia Appendix 1 [file jmir_v25i1e42563_app1.docx]

**Appendix 1: List of chronic conditions**
Twenty-eight chronic conditions and diagnoses were defined using International Statistical Classification of Disease (ICD) 10 codes, selected based on prior VA research. ^19–21^ Chronic conditions include: Acid Related Diseases, Cancers (all types), Alzheimer’s Disease, Arthritis, Asthma, Chronic Obstructive Pulmonary Disease, Heart Failure, Diabetes, HIV/AIDS, Headache, Hepatitis C, Hypertension, Ischemic Heart Diseases, Lower Back Pain, Multiple Sclerosis, Parkinson’s Disease, Peripheral Vascular Disease, Pneumonia, Prostatic Hyperplasia, Renal Failure, Spinal Cord Injury, Stroke, Dementia, Traumatic Brain Injury. Mental health conditions include substance use disorders, severe mental illness (Bipolar disorder, severe depression, and additional psychotic disorders), depression, and post-traumatic stress disorder (PTSD).
