# Supplementary material for: Overcoming Access Barriers for Veterans: Cohort Study of the Distribution and Use of Veterans Affairs’ Video-Enabled Tablets Before and During the COVID-19 Pandemic
Source: J Med Internet Res. 2023 Jan 26;25:e42563. doi: 10.2196/42563 (PMC9912147; doi:10.2196/42563)
Supplement: Multimedia Appendix 2 [file jmir_v25i1e42563_app2.docx]

**Appendix 2: List of mental health conditions**
Mental Health conditions were defined using VA Program Evaluation Resource Center definitions and include substance use disorders, severe mental illness (Bipolar disorder, severe depression, and additional psychotic disorders), depression, and post-traumatic stress disorder (PTSD).
