# Supplementary material for: Overcoming Access Barriers for Veterans: Cohort Study of the Distribution and Use of Veterans Affairs’ Video-Enabled Tablets Before and During the COVID-19 Pandemic
Source: J Med Internet Res. 2023 Jan 26;25:e42563. doi: 10.2196/42563 (PMC9912147; doi:10.2196/42563)
Supplement: Multimedia Appendix 3 [file jmir_v25i1e42563_app3.docx]

**Appendix 3: Description of VA enrollment priority group**

In our evaluation, we use a variable from VA enrollment records called the VA priority group. This variable may be introducing some misclassification bias to the analysis as Veterans are enrolled into the highest group (1 is highest) they qualify for resulting in non-mutually exclusive categorization. For example, because the low-income group is group 5 and disability rating place a Veteran in groups 4 or higher, Veterans who have a larger than a 10% service rated disability will be classified in a higher priority group regardless of their income status. Due to this ranking, the low-income category will not contain all Veterans who are low-income, but will only contain Veterans with low-income. Additionally, Veterans with military honors (e.g., Purple Heart or Medal of Honor) are placed in the highest two priority groups regardless of their disability or income rating which may introduce measurement error and bias when examining disability. The reference group for priority group are Veterans who do not meet any of the criteria for inclusion in the higher ranked groups. Therefore, the rates of the low-income group and disability categories will be attenuated towards the null and may underestimate the true association.
