# Supplementary figures and images for: Overcoming Access Barriers for Veterans: Cohort Study of the Distribution and Use of Veterans Affairs’ Video-Enabled Tablets Before and During the COVID-19 Pandemic
Source: J Med Internet Res. 2023 Jan 26;25:e42563. doi: 10.2196/42563 (PMC9912147; doi:10.2196/42563)

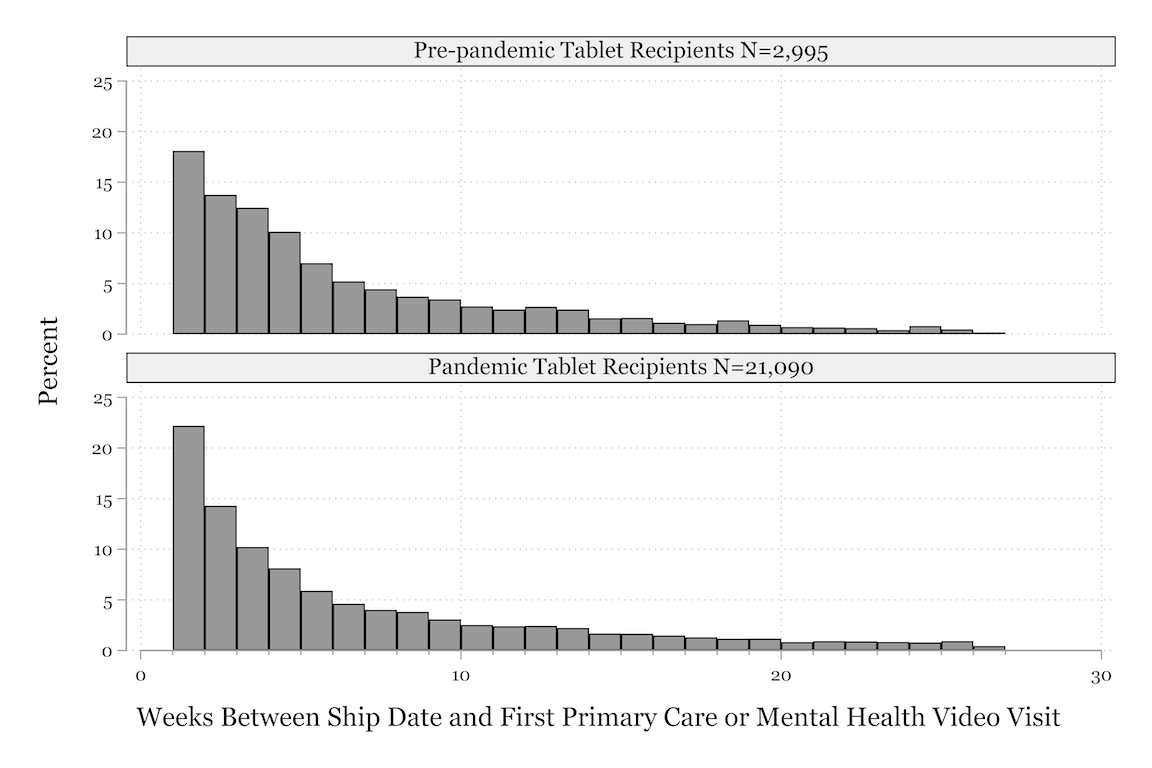

Supplement: Multimedia Appendix 4 [file jmir_v25i1e42563_app4.png]

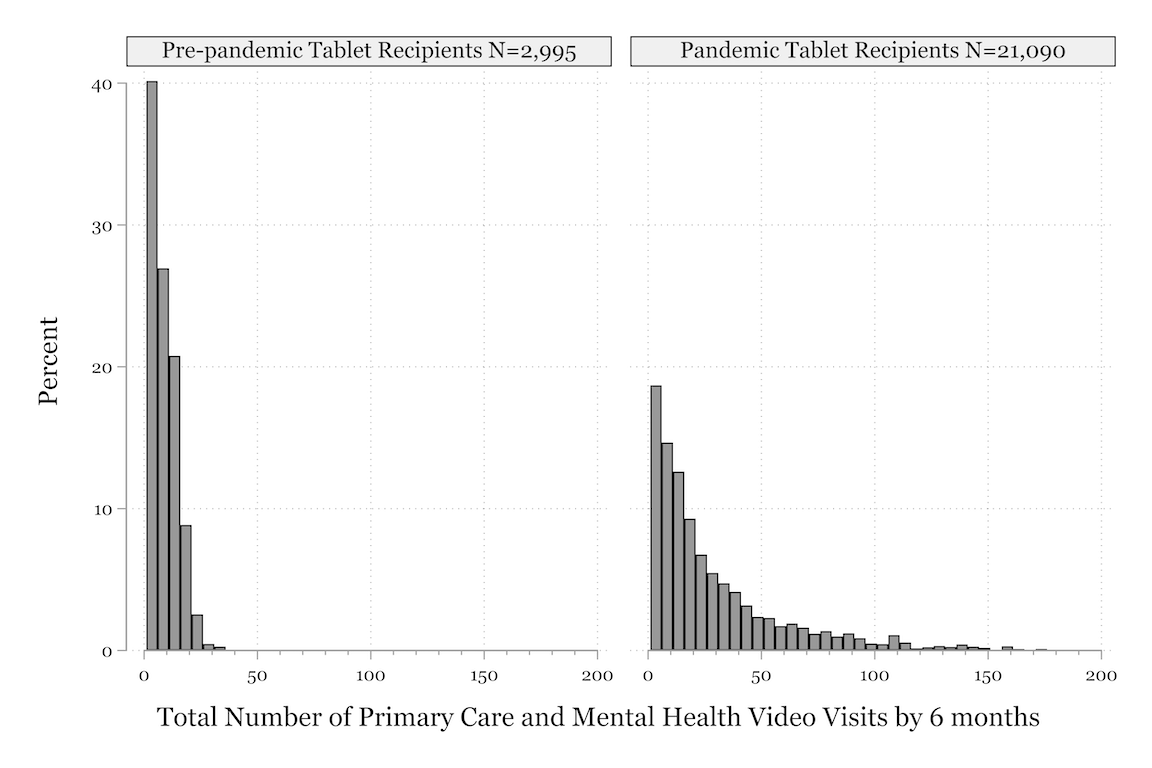

Supplement: Multimedia Appendix 5 [file jmir_v25i1e42563_app5.png]
